# Altered CD161^bright^ CD8^+^ Mucosal Associated Invariant T (MAIT)-Like Cell Dynamics and Increased Differentiation States among Juvenile Type 1 Diabetics

**DOI:** 10.1371/journal.pone.0117335

**Published:** 2015-01-27

**Authors:** Robert Z. Harms, Kristina M. Lorenzo, Kevin P. Corley, Monina S. Cabrera, Nora E. Sarvetnick

**Affiliations:** 1 Department of Surgery-Transplant, University of Nebraska Medical Center, Omaha, Nebraska, United States of America; 2 Pediatric Endocrinology, Children’s Hospital & Medical Center, Omaha, Nebraska, United States of America; 3 Nebraska Regenerative Medicine Program, University of Nebraska Medical Center, Omaha, Nebraska, United States of America; Children’s Hospital Boston/Harvard Medical School, UNITED STATES

## Abstract

Type 1A diabetes (T1D) is believed to be caused by immune-mediated destruction of β-cells, but the immunological basis for T1D remains controversial. Microbial diversity promotes the maturation and activation of certain immune subsets, including CD161^bright^ CD8^+^ mucosal associated invariant T (MAIT) cells, and alterations in gut mucosal responses have been reported in type 1 diabetics (T1Ds). We analyzed T cell populations in peripheral blood leukocytes from juvenile T1Ds and healthy controls. We found that proportion and absolute number of MAIT cells were similar between T1Ds and controls. Furthermore, while MAIT cell proportions increased with age among healthy controls, this trend was not observed among long-standing T1Ds. Additionally, the CD27- MAIT cell subset is significantly increased in T1Ds and positively correlated with HbA1c levels. However, after T1Ds are stratified by age, the younger group has significantly increased proportions of CD27- MAIT cells compared to age-matched controls, and this proportional increase appears to be independent of HbA1c levels. Finally, we analyzed function of the CD27- MAIT cells and observed that IL-17A production is increased in CD27- compared to CD27^+^ MAIT cells. Overall, our data reveal disparate MAIT cell dynamics between T1Ds and controls, as well as signs of increased MAIT cell activation in T1Ds. These changes may be linked to hyperglycemia and increased mucosal challenge among T1Ds.

## Introduction

Human type 1A diabetes (T1D) is believed to be caused by immune-mediated destruction of insulin-producing β cells within the pancreatic islets. The disease can be loosely defined as a state of chronic hyperglycemia coinciding with detectable autoantibodies targeting any of several islet antigen-associated constituents [1, 2]. Due to the difficulty of synthetically managing insulin levels, T1D is associated with a suite of complications resulting from metabolic dysfunction due to imprecise glucose control [3–5]. Although T1D is comparatively well understood in animal models, the etiology of human disease is relatively unknown in terms of immunological factors precipitating disease onset and islet cell damage. Furthermore, causal triggers have not been identified to acceptably explain the modern phenomenon of increasing disease incidence in multiple regions throughout the globe [6, 7]. While genome-wide association studies have implicated several immune-related factors with the risk of clinical disease [8, 9], such factors are predictive in only a minority of patients [10, 11]. From these results and multiple epidemiological studies [12], it is widely accepted that environmental stimuli play a fundamental role in disease onset, and that the face of disease observed in the clinic may in fact represent heterogeneous ontologies.

Interestingly, several lines of evidence connect gut mucosal responses with T1D, in both the preclinical and clinical phases of disease. Prior to clinical onset, at-risk subjects have been shown to possess altered gut microbiotic networks [13–15], increased intestinal permeability [16], and a perturbed metabolome [17]. Changes in gut microbiota [18–20] and intestinal permeability [21–23] persist into clinical disease, and it has been shown that intestinal tissues from T1D patient show hallmarks of immune activation [24, 25] and altered enterocyte microstructure [23]. It is well known that there is dynamic interplay between gut microbiota, intestinal epithelium, and the immune system, with each component regulating and responding to one another [26, 27]. Microbial diversity promotes the maturation and activation of a number of interacting innate and adaptive immune cell subsets, including several T cell subsets, such as mucosal associated invariant T (MAIT) cells, γδ T cells, and Th17 cells. MAIT cells have been shown to be proinflammatory, microbial-sensing IFN-γ and IL-17-secreting cells in the liver and gut lamina propria [28, 29] and have been implicated in the involvement of several inflammatory and autoimmune disorders [30]. γδ T cells migrate to mucosal surfaces, where they can rapidly respond to pathogens and inflammatory signals [31]. Th17 cells, also found in the intestine, are stimulated by gut microbiota [32] and can participate in the pathogenesis of chronic inflammatory diseases including T1D [33]. While the contribution of dysregulated gut homeostasis to β-cell destruction and pancreatic autoimmunity is being explored, one possible conduit between the pancreas and the gut may be the infusion of proinflammatory factors into the pancreas via pancreatic ducts, thus inciting cellular stress and immune activation leading to tissue damage and leukocyte influx, as suggested by Korsgren and colleagues [34]. Ultimately, these and other insults resulting from constituents derived from the gut could lead to immune activation and autoimmunity.

Because human type 1 diabetes remains controversial etiologically and immunologically [35, 36], we sought to broadly evaluate T cell compartments from type 1 diabetics (T1Ds). Our goal was determine which, if any, T cell compartment is altered among T1D. To complete this goal, we analyzed human peripheral blood leukocytes from T1Ds using multiparameter flow cytometry. We designed our flow cytometry investigation to sample a wide swath of T cell subsets. By characterizing the responses of multiple subsets, we wanted to maximize our ability to observe appreciable differences in the immunological terrain of type 1 diabetes. With this information, we hoped to identify populations that may represent the outcome of pathological processes or indicate potential environmental responses that may be contributing to disease. These populations could then be used for further mechanistic studies among diagnosed T1Ds, as well as in the evaluation of prediabetes among at-risk individuals. Here, we present a portion of our findings from these investigations. Our analysis of human MAIT cells from T1Ds and healthy controls revealed disparate population dynamics as well as increased proportions of differentiated CD27^-^ MAIT cells among diabetics. Further analysis of the CD27^-^ population revealed that younger type 1 diabetics possessed increased proportions of this subset compared to age-matched controls. These results suggest increased activation of MAIT cells among T1Ds compared to controls, which may indicate increased mucosal challenge.

## Methods

### Patient population information and sample processing

This work on human subjects was approved by the University of Nebraska Medical Center Institutional Review Board (IRB), protocol #107-09-EP. Informed, written consent using an IRB-approved consent form was documented and obtained from participants and their family or legal guardians prior to participation in the study. Peripheral venous blood was obtained from diagnosed type 1 diabetics (presence of ≥1 autoantibody) and healthy, age-matched controls without history of autoimmune disorders. Patient data are presented in Table 1. Blood was held overnight (<18hours) in EDTA-coated BD vacutainers prior to lysis and surface staining. We lysed red blood cells using ammonium chloride lysis buffer and calculated white blood cells per milliliter (ml) using a haemocytometer. To label dead cells prior to flow cytometric antibody staining, we utilized LIVE/DEAD Fixable Dead Cell Stain (life technologies) according to manufacturer’s protocol. After LIVE/DEAD labeling, cells were washed twice with PBS, and then resuspended in a flow cytometry staining buffer (FCSB) comprised of PBS, 0.75% BSA, 0.05% sodium azide, and 1mM EDTA. We then blocked Fc receptors using irrelevant unlabeled human IgG. After Fc block, cells were incubated with antibody cocktails for 30 minutes at 4ºC in the dark. The combination of fluorochrome-conjugated antibodies we used in this flow cytometry panel are shown in Table 2. We then washed the cells twice with FCSB and incubated cells with streptavidin-conjugated fluorochomes for 20 minutes at 4ºC in the dark. Cells were then washed twice and fixed with 3% paraformaldehyde (PFA) for 20 minutes at room temperature in the dark. Cells were then washed once with FCSB, resuspended in FCSB, and analyzed on LSR II flow cytometer (BD Biosciences) within 24 hours.

**Table 1:** Patient population data.

	**Control**	**T1D** *	**NT1D** *	**LT1D** *
n (n female)*	35 (19)	31 (12)	18 (7)	13 (5)
Mean age in years (range)	10.2 (2–17)	10.0 (2–16)	9.3 (2–16)	11.0 (4–16)
Mean disease duration in months (range)	N/A	14.4 (0–57)	4.5 (0–11)	28.1 (14–57)
Mean HbA1c at draw (range)	N/A	8.2 (5.55–15)	8.7 (5.5–15)	7.4 (6–9.4)

* T1D—type 1 diabetics; NT1D—new-onset type 1 diabetics (<12 months since diagnosis); LT1D—long-standing type 1 diabetics (≥ 12 months since diagnosis); n (n female)—total number of patients per group, n, and number of patients which are female (n female)

**Table 2:** List of primary antibodies and other reagents in the flow cytometry panel used to generate the data from type 1 diabetics and controls presented in this study.

***Antibody***	***Clone***	***Fluorochrome***	***Source***
CCR7	3D12	PE	eBioscience
CD3ε	HIT3a	Alexa Fluor 700	BioLegend
CD4	RPA-T4	BD Horizon^V500^	BD Biosciences
CD8	3B5	QDot705	life technologies
CD27	O323	APC-Cy7	BioLegend
CD45	HI30	PerCP	BioLegend
CD45RA	MEM-56	PE-Texas Red	life technologies
CD57	HCD57	APC	BioLegend
CD62L	MEL-14	eFluor 605NC	eBioscience
CD127 (IL-7Rα)	A019D5	Brilliant Violet 421	BioLegend
CD161	HP-3G10	FITC	BioLegend
CD218a (IL-18Rα)	H44	Biotin	BioLegend
HLA-DR	L243	PE-Cy7	BioLegend
***Other***		***Fluorochrome***	***Source***
Streptavidin		QDot655	life technologies
LIVE/DEAD Fixable Dead Cell Stain		UV Blue	life technologies

### Data analysis and T cell gating

**Fig 1:**
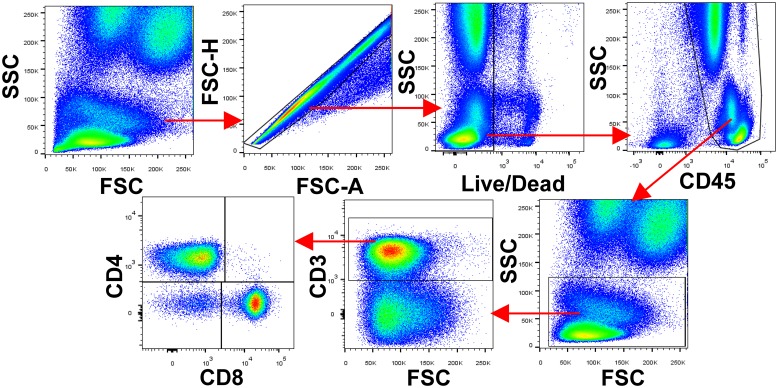
Depiction of gating strategy used to identify CD8 T cells in this study. We identified T cells from total leukocytes as shown. We first selected singlets using forward scatter area (FSC-A) versus forward scatter height (FSC-H) parameters. From the singlet population, we removed dead cells from total events by gating on LIVE/DEAD^-^ events. Then, we selected total leukocytes from the living, singlet population using the pan-leukocyte marker CD45. To proceed to T cell selection, we gated on lymphocytes and monocytes based upon FSC and side scatter (SSC) properties. We defined T cells using the expression level of the pan-T cell marker CD3. Finally, we split T cells into CD4, CD8 and double negative (DN) populations using CD4 and CD8 expression as shown. In summary, events defined as CD8^+^, CD4^-^, CD3^+^, CD45^+^, LIVE/DEAD^-^, mononuclear singlets were defined as CD8 T cells for this analysis.

We generated scatterplots and analyzed flow cytometry data using FlowJo analysis software (v10, Tree Star, Inc.). Events defined as CD8^+^, CD4^-^, CD3^+^, CD45^+^, LIVE/DEAD^-^, mononuclear singlets were defined as CD8 T cells for this analysis. See Fig. 1 for an illustrated gating strategy.

### Intracellular flow cytometry

We isolated peripheral blood mononuclear cells (PBMCs) from healthy volunteers (n = 6) using Ficoll-Paque Plus (GE Healthcare). We then cultured PBMCs at 1 × 10^6^ per ml overnight in X-VIVO 15 (Lonza) with 2% human AB serum (Corning Cellgro) and 10 units recombinant human IL-2 (Cell Sciences) per ml. The following morning, we added cell stimulation cocktail plus protein transport inhibitors (eBioscience, item# 00-4975) to our cultured PBMCs to induce cytokine production. At approximately 5.5 hours after the addition of the cell stimulation cocktail, the PBMCs were prepared for flow cytometric staining. Surface staining was performed as described above. Following fixation, the cells were permeabilized using Permeabilization Wash Buffer (BioLegend) according to manufacturer’s recommendations. After blocking cells as described above, we added incubated cells with fluorochrome-conjugated antibodies targeting intracellular proteins for 30 minutes at 4ºC in the dark. The cells were then washed twice with Permeabilization Wash Buffer, twice with FCSB, and resuspended in FCSB for analysis as above. The antibodies used for intracellular staining are shown in Table 3.

**Table 3:** List of primary antibodies and other reagents utilized in the flow cytometry panel used to generate the data on IL-17A expression from healthy controls presented in this study.

***Antibody***	***Clone***	***Fluorochrome***	***Source***
CD3ε	OKT3	PerCP-Cy5.5	Tonbo Biosciences
CD8α	RPA-T8	Brilliant Violet 786	BD Biosciences
CD27	0323	PE-Cy7	BioLegend
CD161	HP-3G10	PE	BioLegend
IL-17A	BL168	Alexa Fluor 647	BioLegend
***Other***		***Fluorochrome***	***Source***
LIVE/DEAD Fixable Dead Cell Stain		UV Blue	life technologies

### Statistics

Using FlowJo, we calculated the percent of parent, percent of CD8 T cells, percent of total T cells, and percent of total leukocytes. The number of circulating cells per milliliter (ml) was calculated by dividing the percent of total leukocytes by 100, then multiplying this value by the number of total white blood cells per ml as calculated above. To determine proportional and numerical differences between subsets for all cohorts, we tested for statistical significance using the Mann-Whitney U Test. For correlation analysis, we first transformed immune subset data to logarithmic values. We then performed Pearson product-moment correlation coefficient (Pearson’s r) analysis to test for strength of correlations. We further performed linear regression on our correlations to generate trend lines and to compare correlations between experimental groups. For all statistical tests, results were considered significant when p < 0.05. We used GraphPad Prism v6 to perform statistical tests and to create figures.

## Results

### The proportion of CD27^-^ MAIT cells is significantly increased in juvenile type 1 diabetics

**Fig 2:**
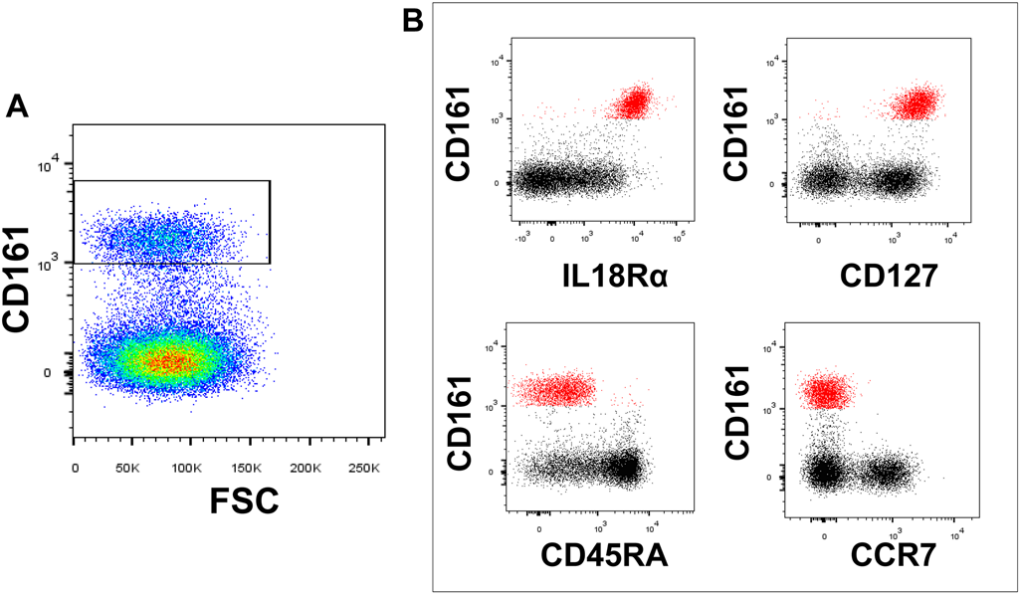
Gating of CD161^bright^ CD8 T cells and phenotype suggesting mucosal associated invariant T (MAIT) cell status. **A**. Representative gating of CD161^bright^ events among the CD8 T cell compartment. **B**. CD161^bright^ CD8 T cells expressed high levels of IL-18Rα and CD127 and negligible levels of CD45RA and CCR7. Combined, this phenotype describes MAIT cells.

**Fig 3:**
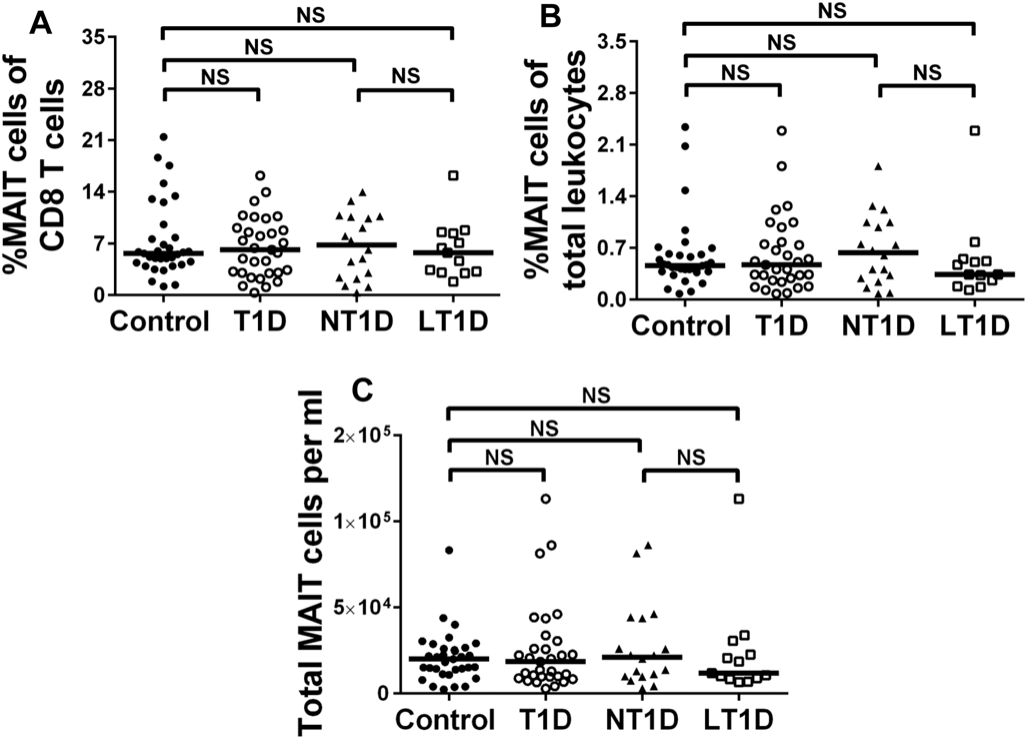
Controls and diabetics harbor similar proportions and numbers of CD161^bright^ CD8 mucosal associated invariant T (MAIT) cells. The proportion of MAIT cells among total CD8 T cells (**A**.) and among total leukocytes (**B**.) is similar between controls and type 1 diabetics (T1D). **C**. Controls and diabetics also possess similar numbers of MAIT cells per ml of blood. Stratifying our type 1 diabetics cohort into new-onset (NT1D, <12 months since diagnosis) and long-standing (LT1D, ≥12 months since diagnosis) diabetics also revealed no differences compared to controls (**A—C**). Significance was determined using the Mann-Whitney U Test. Bars represent median. NS = not significant.

**Fig 4:**
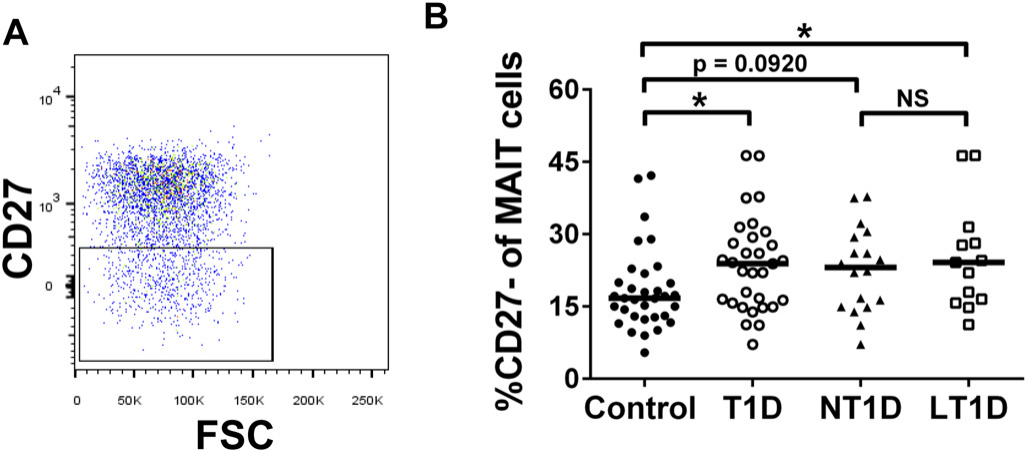
Type 1 diabetics possess increased proportions of CD27^-^ MAIT cells compared to controls. **A**. Representative gating of CD27^-^ events among the MAIT cell compartment. **B**. Type 1 diabetics (T1D) have significantly increased proportions of CD27^-^ MAIT cells compared to controls. Stratifying the type 1 diabetic cohort into new-onset (NT1D, <12 months since diagnosis) and long-standing (LT1D, ≥12 months since diagnosis) revealed a significant increase among the LT1D population, with a trend toward significance among the NT1D population. Significance was determined using the Mann-Whitney U Test. Bars represent median. * = p < 0.05, NS = not significant.

It has been hypothesized that an imbalanced intestinal flora is an environmental trigger that may promote aberrant mucosal immune responses and contribute to the development of T1D [37]. Within the CD8 T cell compartment resides a subset of cells expressing high levels of CD161 (CD161^bright^, Fig. 2A), which are chiefly mucosal associated invariant T (MAIT) cells [28, 38]. MAIT cells possess a semi-invariant T cell receptor and can be further phenotypically identified by expression of high levels of CD127 and IL-18Rα and low levels of CCR7 and CD45RA (Fig. 2B). We reasoned that if T1Ds suffered from altered intestinal immunity, we may observe altered proportions and absolute numbers of MAIT cells. However, this was not the case, as we observed that diabetics and controls possessed similar proportions and numbers of MAIT cells (Fig. 3A–C). We were also curious if MAIT cell alterations may be associated with disease duration. Within the first year following diagnosis, T1Ds have demonstrated variability among physiological parameters [39–41], and this variability could influence immunological subsets. Therefore, we stratified our diabetic cohort into new-onset (NT1D, <12 months since diagnosis) and long-standing (LT1D, ≥12 months since diagnosis) subsets. These stratifications did not reveal significant differences among MAIT cells (Fig. 3A–C). We further analyzed the MAIT cell compartment for expression of the homing marker CD62L and costimulatory marker CD27. Although we observed no change in proportion or number CD62L^+/-^ MAIT cell subsets (data not shown), we did observe that T1Ds possessed a reduced proportion of CD27^+^ MAIT cells (data not shown) with a corresponding increase in proportion of CD27^-^ MAIT cells compared to controls (Control vs. T1D, p = 0.0224; Control vs. LT1D, p = 0.0418; Fig. 4A & B).

### The dynamics of MAIT cell proportion associated with aging are altered among juvenile type 1 diabetics

Human studies have suggested that the circulating MAIT cell population is present in cord blood and increases in proportion sometime between infancy and adulthood [28, 38], but then declines with increased age [42]. To our knowledge, the developmental dynamics of MAIT cells have not been investigated among human diabetic juveniles, nor is it entirely clear if MAIT cells increase in proportion steadily or abruptly over time among healthy juveniles. To answer these questions, we tested for correlations between MAIT cells and age of donor among our experimental groups. We first transformed our proportional data into logarithmic values and performed Pearson product-moment correlation coefficient (Pearson’s r) analysis to test for strength of correlations. We then performed linear regression on our correlations to generate trend lines and to compare correlations between experimental groups.

**Fig 5:**
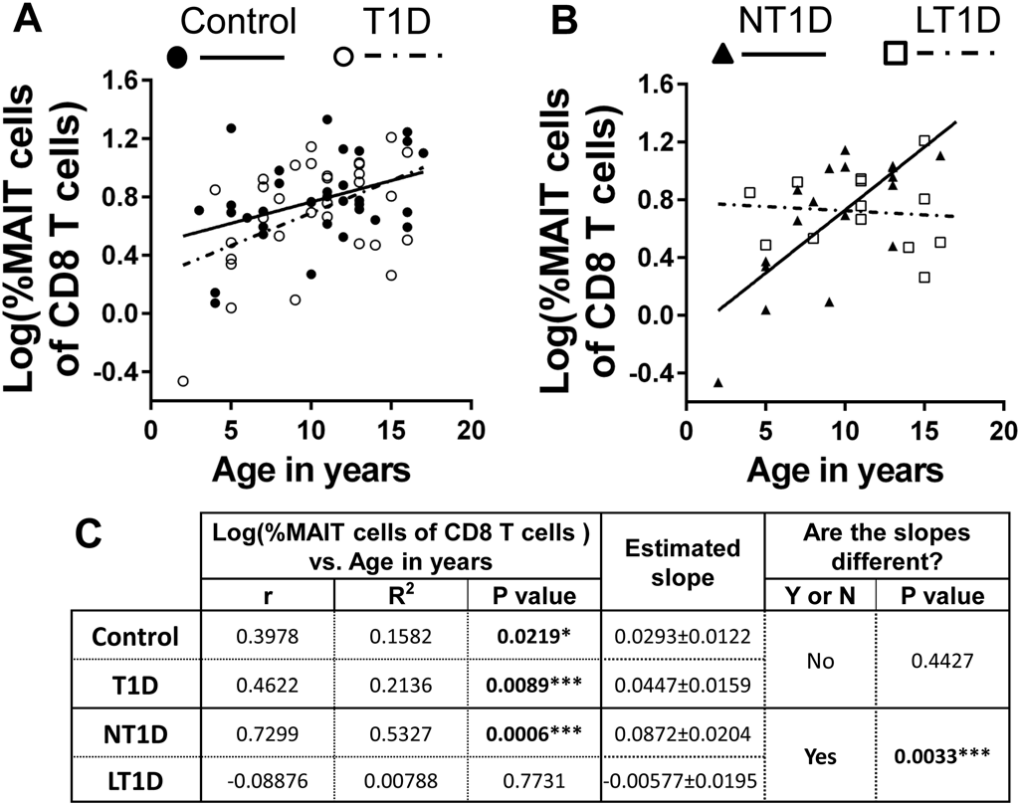
The proportion of MAIT cells of total CD8 T cells is significantly and positively correlated with age in years among controls, total type 1 diabetics (T1D), and new-onset diabetics (NT1D), but not among long-standing diabetics (LT1D). **A**. Correlation of log(%MAIT cells of total CD8 T cells) versus age in years among controls and total type 1 diabetics (T1D). Controls values are represented by solid circles and a solid trend line. Values for T1D are represented by open circles and a dotted trend line. **B**. Correlation of log(%MAIT cells of total CD8 T cells) versus age in years among new-onset T1D (NT1D) and long-standing T1D (LT1D). Values for NT1D are represented by solid triangles and a solid trend line. Values for LT1D are represented by open squares and a dotted trend line. **C**. Results of Pearson’s r analysis and linear regression. * = p<0.05, ** = p <0.01, *** = p<0.001

**Fig 6:**
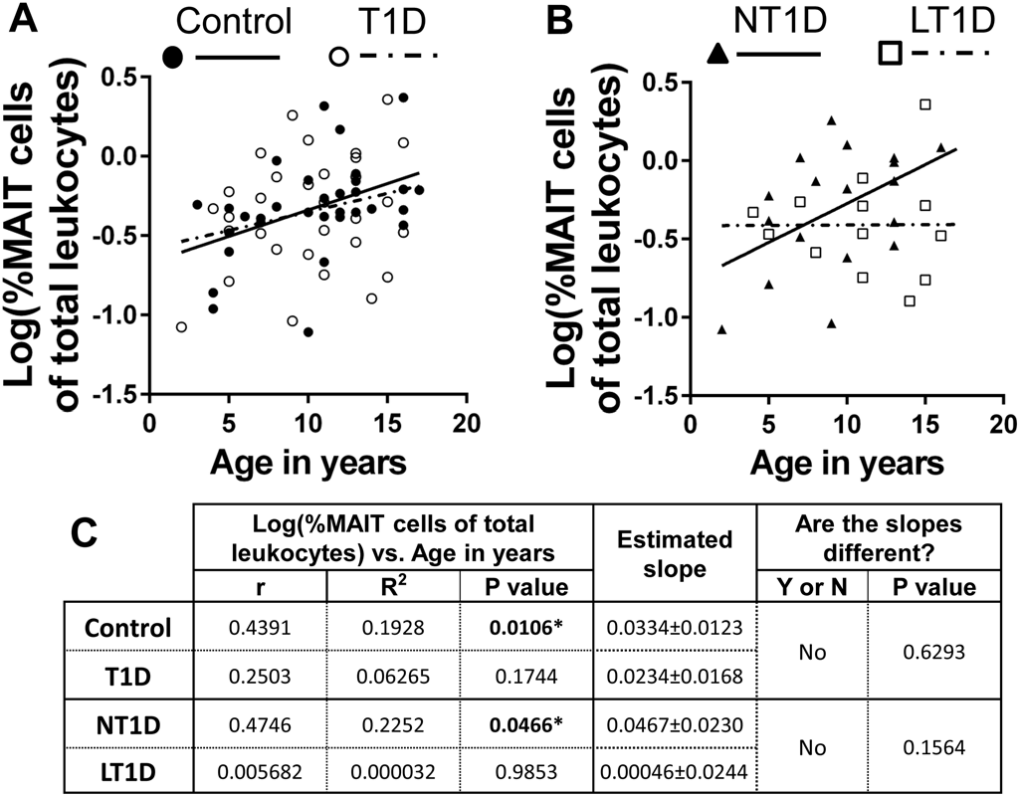
The proportion of MAIT cells of total leukocytes is significantly and positively correlated with age in years among controls and new-onset diabetics (NT1D), but not total type 1 diabetics (T1D) or long-standing diabetics (LT1D). **A**. Correlation of log(%MAIT cells of total leukocytes) versus age in years among controls and total type 1 diabetics (T1D). Controls values are represented by solid circles and a solid trend line. Values for T1D are represented by open circles and a dotted trend line. **B**. Correlation of log(%MAIT cells of total leukocytes) versus age in years among new-onset T1D (NT1D) and long-standing T1D (LT1D). Values for NT1D are represented by solid triangles and a solid trend line. Values for LT1D are represented by open squares and a dotted trend line. **C**. Results of Pearson’s r analysis and linear regression. * = p<0.05

Using these approaches, we noted a significant positive correlation between age of donor and proportion of MAIT cells of total CD8 T cells among controls and total type 1 diabetics (Fig. 5A & C). Interestingly, when we examined new-onset (NT1D) and long-standing diabetics (LT1D) separately, we observed that while the NT1Ds possessed a robust, significant positive correlation between age and proportion of MAIT cells of CD8 T cells, this correlation was not significant among LT1Ds and demonstrated little sign of increasing with time (Fig. 5B–C). Subsequent analysis revealed that these correlations were significantly different (Fig. 5C). We observed similar results when we examined the proportion MAIT cells of total T cells (data not shown). However, upon examination of the proportion MAIT cells of total leukocytes, we observed that the strength of the correlation among total diabetics decreased and lost significance and that the correlations between the new-onset and long-standing populations were no longer significantly different (Fig. 6A–C). In contrast, the control population demonstrated a significant positive correlation between age in years and proportion MAIT cells of total leukocytes (Fig. 6A & C). These results demonstrate that MAIT cells increase steadily in proportion over time from infancy into young adulthood among healthy controls. This increase in proportion was observable among total CD8 T cells, total T cells and total leukocytes. Type 1 diabetics revealed similar increases in proportion with age among total CD8 T cells and total T cells, however, the correlation weakened and lost significance when comparing proportion of total leukocytes. By comparing new-onset and long-standing diabetics, we observed that MAIT cells among the long-standing group demonstrated little sign of increasing in proportion with age in stark opposition to the robust positive correlation observed among the new-onset group. This suggests disparate homeostatic dynamics among MAIT cells influenced by disease duration.

**Fig 7:**
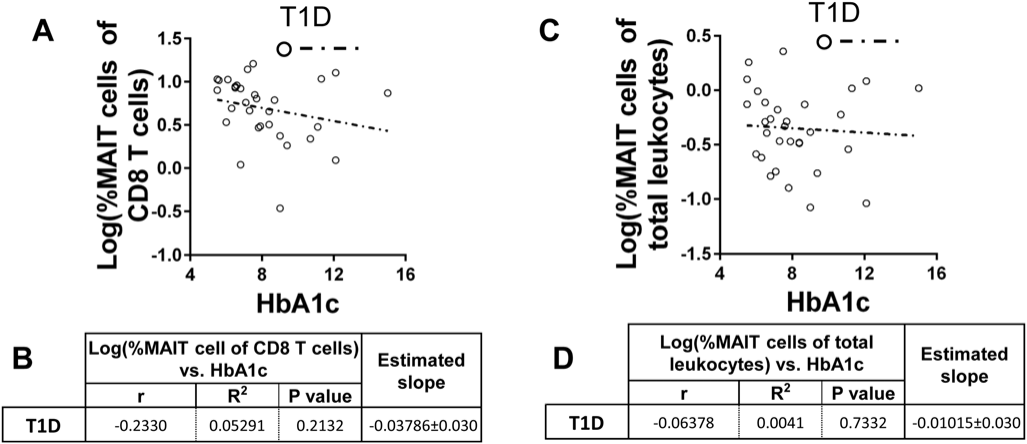
The proportion of MAIT cells of both CD8 T cells and total leukocytes is negatively but not significantly correlated with HbA1c among type 1 diabetics (T1D). **A**. Correlation of log(%MAIT cells of CD8 T cells) versus HbA1c among total T1D. **B**. Results of Pearson’s r analysis and linear regression. **C**. Correlation of log(%MAIT cells of total leukocytes) versus HbA1c among total T1D. **D**. Results of Pearson’s r analysis and linear regression. For both A and C, open circles and dotted trend line represent T1D.

From the observation that MAIT cells are not expanded among LT1Ds, we reasoned that proportion of MAIT cells may be altered with time since diagnosis or hyperglycaemia. We did not observe strong correlations between MAIT proportions and time since diagnosis among diabetics (S1 File). Interestingly, correlation analysis revealed an upward trend with time since diagnosis among the new-onset cohort, while the LT1D group exhibited a downward trend. However, these correlations were not statistically significant. Finally, we did not observe a significant correlation with glycated hemoglobin and MAIT cell proportion (Fig. 7). Unfortunately, we were unable to investigate a correlation with c-peptide due to an insufficient number of patients possessing c-peptide values at blood draw.

### Younger type 1 diabetics possess greater proportions of CD27^-^ MAIT cells than older type 1 diabetics

**Fig 8:**
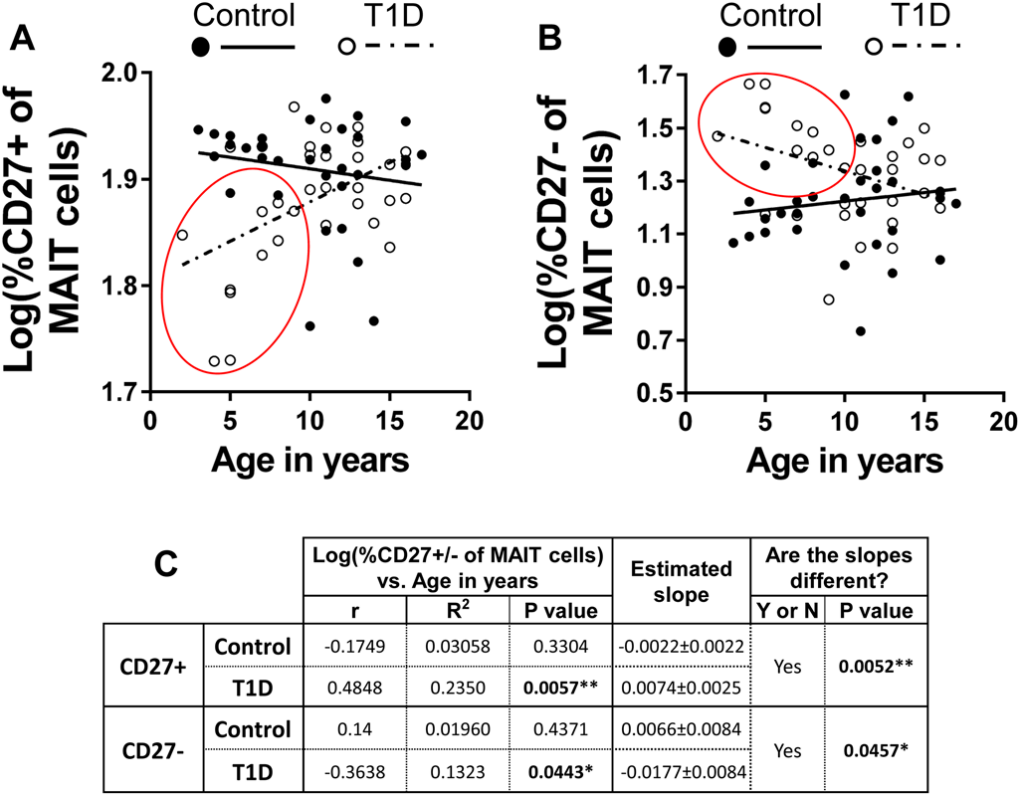
In contrast to controls, the proportion CD27^+^ of MAIT cells is significantly and positively correlated with age among total type 1 diabetics (T1D), while the proportion CD27^-^ of MAIT cell proportion is significantly and negatively correlated with age among T1D. **A**. Correlation of log(%CD27^+^ of MAIT cells) versus age in years among controls and T1D. Controls values are represented by solid circles and a solid trend line. Values for T1D are represented by open circles and a dotted trend line. **B**. Correlation of log(%CD27^-^ of MAIT cells) versus age in years among controls and T1D. Controls values are represented by solid circles and a solid trend line. Values for T1D are represented by open circles and a dotted trend line. **C**. Results of Pearson’s r analysis and linear regressions for CD27^+^ and CD27^-^ MAIT cells. * = p<0.05, ** = p <0.01

Our data revealing altered proportions of CD27 expression by MAIT cells among diabetics suggested there may be developmental differences among these populations over time. We investigated this hypothesis by performing correlation analysis between proportion of CD27^+^ or CD27^-^ MAIT cells of CD8 T cells and age of donor. The control group revealed a steady decrease of CD27^+^ MAIT cells and increase of CD27^-^ MAIT cells with age, though neither correlation was significant (Fig. 8A–C). In sharp contrast, total T1Ds exhibited a significant increase in proportion of CD27^+^ MAIT cells with age of donor, while proportion of CD27^-^ MAIT cells significantly decreased with age of donor (Fig. 8A–C). Analysis of the correlations revealed that the trends between the controls and diabetics were significantly different for both CD27^+^ and CD27^-^ MAIT subsets (Fig. 8C).Correlation analysis between proportion of CD27^+^ or CD27^-^ MAIT cells of CD8 T cells and age of donor for type 1 diabetics divided into new-onset or long-standing subsets revealed comparable trends to those seen in Fig. 8 (S2 File).

**Fig 9:**
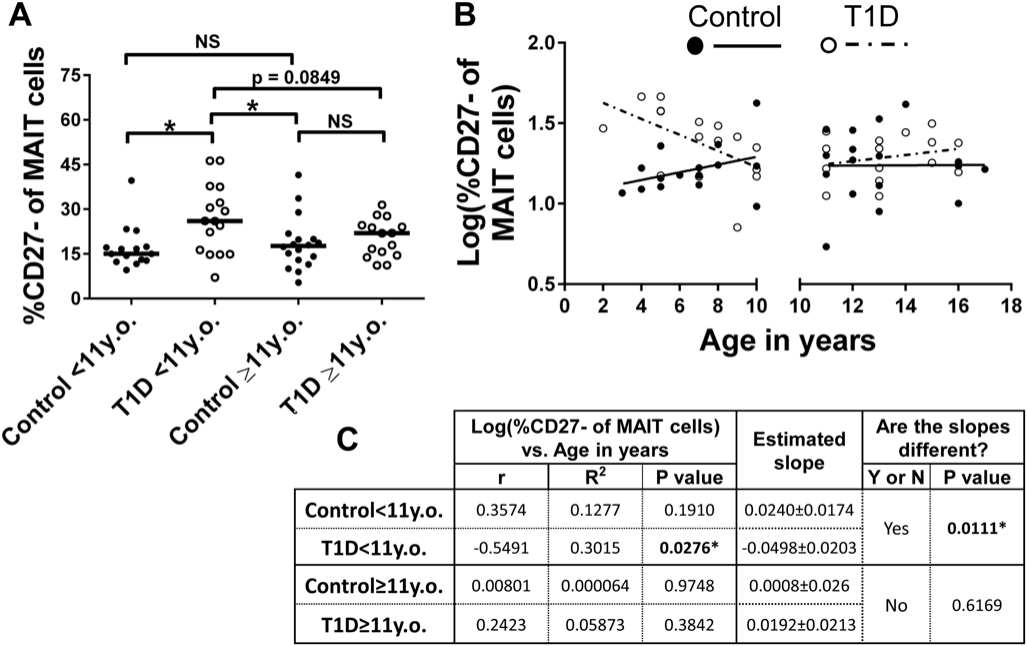
Younger type 1 diabetics (T1D) possess increased proportions of CD27^-^ MAIT cells compared to age-matched controls. **A**. T1D <11 years old (y.o.) have significantly increased proportions of CD27^-^ MAIT cells compared to age-matched controls (Control<11y.o.) and older controls (Control≥11y.o.), and approach a significant increase compared to T1D≥11 y.o. Significance was determined using the Mann-Whitney U Test. Bars represent median, * = p < 0.05, NS = not significant. **B**. Correlation of log(%CD27^-^ of MAIT cells) versus age in years splitting controls and type 1 diabetics at < 11 years of age and ≥ 11 years of age. **C**. Results of Pearson’s r analysis and linear regression. * = p<0.05

After analyzing the correlation data in Fig. 8, we observed that younger T1Ds appeared to possess increased proportions of CD27^-^ MAIT cells (and, conversely, decreased proportions of CD27^+^ MAIT cells) compared to controls (red circles on Fig. 8), suggesting maturational or activation differences between younger and older type 1 diabetics and controls. To investigate this further, we stratified our data into 4 subsets: healthy controls of less than 11 years of age (Control<11y.o.), type 1 diabetics of less than 11 years of age (T1D<11y.o.), healthy controls of 11 years of age or more (Control≥11 y.o.) and type 1 diabetics of 11 years of age or more (T1D≥11y.o.). These age-based, synthetic stratifications roughly partition children and pre-adolescents from adolescents and teenagers. Demographic and physiological data for these stratifications are presented in Table 4. Next, we compared these subsets for proportional differences among CD27^-^ MAIT cells. This comparison revealed that our cohort of T1D<11y.o. possessed significantly increased proportions of CD27^-^ MAIT cells compared to Control<11y.o. (p = 0.012) and Control≥11 y.o. (p = 0.038) groups (Fig. 9). Follow-up correlation analysis on these four cohorts revealed a significant negative correlation between increasing age and proportion of CD27^-^ MAIT cells among T1D<11y.o., while Control<11y.o. possessed a positive though insignificant trend for the same parameters (Fig. 9B & C). Comparison of linear regression models for Control<11y.o. and T1D<11y.o. yielded a significant difference between the two models (Fig. 9C). Our correlation analysis and comparison of the linear regressions of the T1D≥11y.o. and Control≥11 y.o. groups yielded correlations that were not significant for either group and slopes that were not significantly different (Fig. 9C). Combined, these data demonstrate that younger diabetics have increased proportions of CD27^-^ MAIT cells compared to healthy, age-matched controls. The mechanistic basis for these differences is not yet clear. However, MAIT cells have been shown to be uniquely activated by riboflavin metabolites and precursors that are derived from specific bacteria and yeasts [43]. Thus, alterations within this compartment could be directly related to microbiotic differences between diabetics and controls, as has been demonstrated previously [13–15].

**Table 4:** Patient population data after age stratification.

	Control <11 y.o.*	T1D* <11 y.o.	**Control ≥11 y.o.**	**T1D ≥11 y.o.**
n (n female)*	15 (9)	16 (7)	18 (9)	15 (5)
Mean age in years (range)	6.3 (3–10)	6.9 (2–10)	13.3 (11–17)	13.3 (11–16)
Mean disease duration in months (range)	N/A	11.6 (0–57)	N/A	17.4 (0–40)
NT1D/LT1D*	N/A	12/4	N/A	6/9
Mean HbA1c at draw (range)	N/A	8.3 (5.5–15)	N/A	8.1 (5.5–12.1)

* T1D—type 1 diabetics; y.o.—years old; n (n female)—total number of patients per group, n, and number of patients which are female (n female); NT1D/LT1D—ratio of new-onset type 1 diabetics (NT1D, <12 months since diagnosis) to long-standing type 1 diabetic (LT1D, ≥12 months since diagnosis)

### The proportion of CD27^-^ MAIT cells is associated with HbA1c levels among juvenile type 1 diabetics

**Fig 10:**
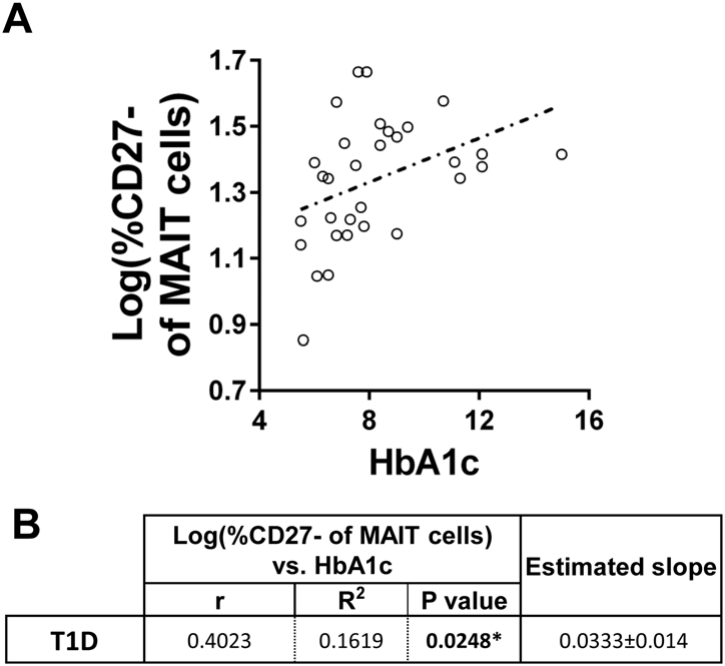
The proportion CD27^-^ of MAIT cells is significantly and positively correlated with HbA1c among total type 1 diabetics (T1D). **A**. Correlation of log(%CD27^-^ of MAIT cells) versus HbA1c among T1D. Values for T1D are represented by open circles and a dotted trend line. **B**. Results of Pearson’s r analysis and linear regression. * = p<0.05

**Fig 11:**
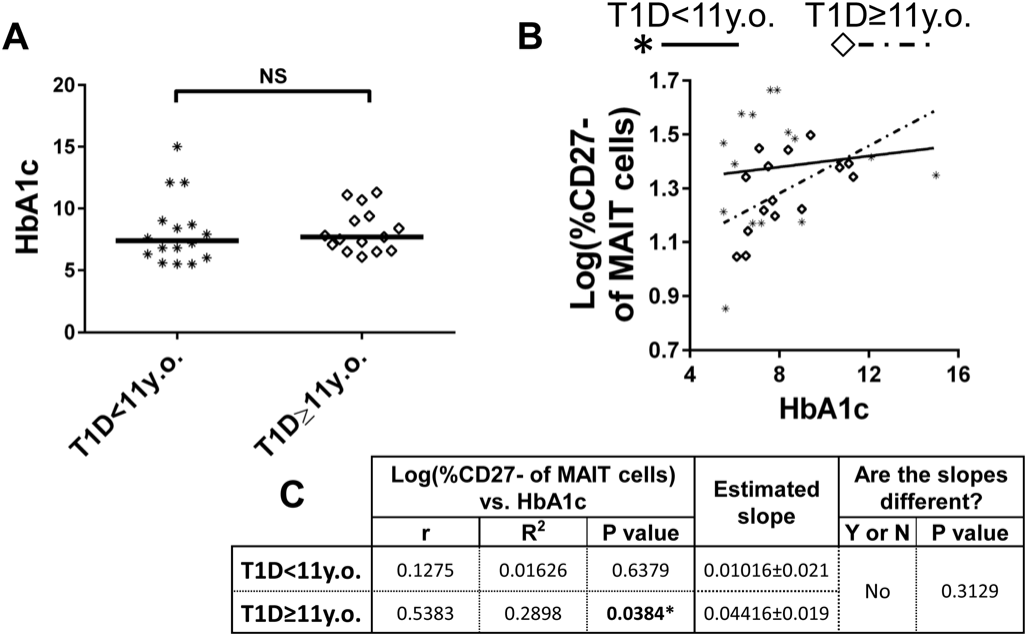
HbA1c levels are similar between age-stratified type 1 diabetic (T1D) populations, yet HbA1c is positively and significantly correlated with CD27^-^ proportions among older T1D (≥11 years old (y.o.)) and not with younger T1D (<11 years old (y.o.)). **A**. Comparison of HbA1c levels between T1D<11 y.o. and T1D≥ 11 y.o. Significance was determined using the Mann-Whitney U Test. Bars represent median, NS = not significant. **B**. Correlation of log(%CD27^-^ of MAIT cells) versus HbA1c among T1D<11 y.o. and T1D≥ 11 y.o. Values for T1D<11 y.o. are represented by asterisks and a solid trend line, while T1D≥ 11 y.o. are represented by open diamonds and a dotted trend line. **C**. Results of Pearson’s r analysis and linear regression. * = p<0.05

In order to evaluate the contribution of hyperglycaemia to alterations among CD27^-^ MAIT cells, we tested to see if CD27^-^ MAIT cell proportional differences correlated with HbA1c. We observed a significant positive correlation between the two variables among total T1Ds (Fig. 10A & B), indicating that increasing circulating glucose levels may directly or indirectly influence MAIT cell differentiation. Since the proportional expansion of CD27^-^ MAIT cells appeared most pronounced in younger T1Ds, we were curious if this could be explained simply by increased HbA1c levels among this group. However, there was no significant difference in HbA1c levels between older and younger T1Ds (Fig. 11A). Furthermore, while the CD27^-^ MAIT cells proportions were significantly and positively correlated with HbA1c among T1D≥11y.o., this correlation was not significant among T1D<11y.o. (Fig. 11B & C). These intriguing results suggest that although hyperglycaemia may contribute to increased proportions of CD27^-^ MAIT cells among older juveniles, alternate explanations are necessary to fully explain the increased CD27^-^ MAIT cell proportions we observed in T1D<11y.o.

### IL-17A production is substantially greater among the CD27^-^ subset of CD161^bright^ CD8 T cells compared to the CD27^+^ subset

**Fig 12:**
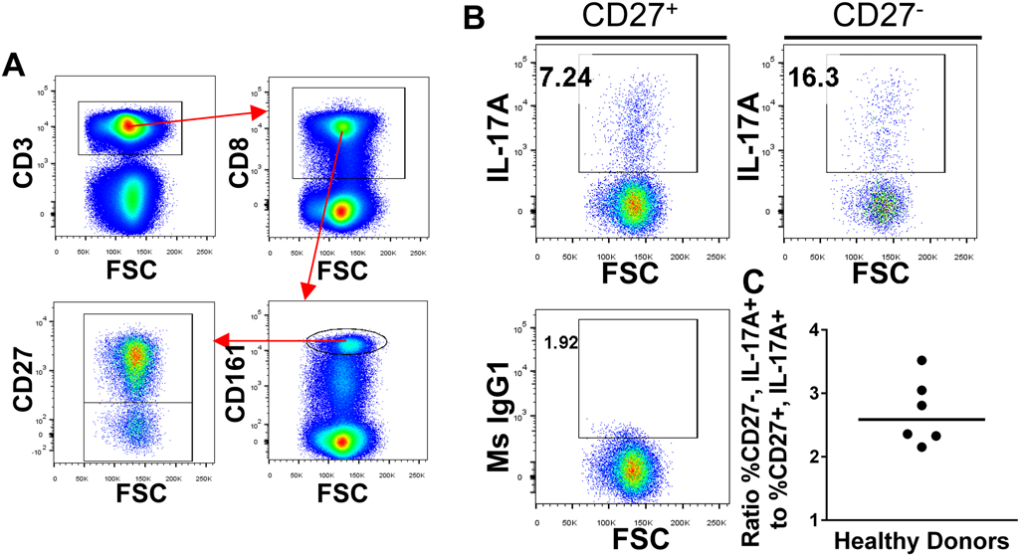
CD27^-^ CD161^bright^ CD8 T cells contain a greater proportion of IL-17A-producing cells than the CD27^+^ subset. PBMCs freshly isolated from healthy human donors (n = 6) were cultured overnight and then stimulated for 5.5 hours in the presence of PMA and ionomycin with protein transport inhibitors. Following stimulation, we performed surface and intracellular staining to quantitate IL-17A expression by CD27^+/-^ CD161^bright^ CD^+^ MAIT cells. **A**. Representative gating of CD27^+/-^ MAIT cells from total stimulated PBMCs. CD3^+^ events were selected from pregated Live/Dead^-^ singlets. **B**. Representative gating showing IL-17A expression on CD27^+^ and CD27^-^ MAIT cells. Gates were set according to background determined by appropriate isotype control. **C**. CD27^-^ MAIT cells contain a greater portion of IL-17A-producing cells than the CD27^+^ MAIT cell subset. Bar represents median.

As stated above, functional differences between CD27^-^ and CD27^+^ MAIT cells have not been investigated. It is known that the MAIT cell compartment contains IL-17A, TNF-α, and IFN-γ producing subsets [28]. To investigate differences among cytokine production by CD27^-^ or CD27^+^ MAIT cells, we performed intracellular flow cytometry on PMA and ionomycin-stimulated peripheral blood mononuclear cells (PBMCs) from healthy donors (n = 6). Our results revealed that neither the CD27^+^ nor the CD27^-^ subsets were consistently polarized towards greater IFN-γ or TNF-α expression, though in all cases the majority of cells from both subsets expressed each cytokine (data not shown). Interestingly, we observed that CD27^-^ MAIT cells consistently possessed a greater proportion (median = 2.6 fold greater) of IL-17A^+^ events compared to CD27^+^ MAIT cells (Fig. 12A & B). These data demonstrated that IL-17A production is enhanced among CD27^-^ MAIT cells. Thus, the loss of CD27 expression on MAIT-like cells appears to denote further differentiation than among the CD27^+^ MAIT compartment, an observation in accordance with that seen among other T cell subsets. These results also suggest that the CD27^-^ MAIT-like subset harbors more IL-17A-producing cells than their CD27-expressing counterparts. Thus, the expansion of CD27^-^ MAIT-like cells observed among T1Ds may likewise harbor a greater proportion of proinflammatory IL-17A producers, a hypothesis which is currently under investigation.

## Discussion

In this investigation, we observed several abnormalities associated with CD161^bright^ MAIT-like CD8 T (MAIT) cells among type 1 diabetics. For example, we observed unique MAIT cell developmental dynamics among type 1 diabetics compared to our control group. Our assessment of human MAIT cells from healthy controls indicates that among non-diabetic conditions, the MAIT cell compartment expands steadily from infancy to young adulthood as implied by previous reports [28, 38]. From birth onward, children can harbor a wide range of gut microbes and the inflation in this population may be indicative of this exposure. Nevertheless, it has recently been shown that a somewhat stable flora assemblage of higher-order taxonomic groups can be seen in children by around 3 years of age [44], correlating with the introduction of solid food. Therefore, if microbiotic species assemblages become proportionately constant early in childhood, it is currently unclear what could be promoting this expansion of MAIT cells over time. One possibility is that increasing mucosal surface area during growth and development creates additional habitable space for microbiota, and a greater proportion of responsive immune cells are needed to address this stimulus. Our data also demonstrate that among healthy controls, this subset is given increased niche space among CD8 T cells, total T cells (total CD3^+^ events), and total leukocytes (total CD45^+^ events: B, T, and NK cells, monocytes and granulocytes). However, this does not appear to be correlated with increased proportions of CD8 T cells, T cells or leukocytes with age among healthy controls or type 1 diabetics (data not shown). Thus, the increased proportion of MAIT cells must come with proportional decreases amongst other leukocyte subsets, and coincide with positive feedback mechanisms for the MAIT cell compartment signaling lineage proliferation and/or survival.

Compared to healthy controls, we observed a similar increased proportion of MAIT cells with increasing age among our type 1 diabetic group among CD8 T cells and T cells, although this correlation was not significant among total leukocytes. Upon stratification of the diabetics into new-onset (<12 months) and long-standing (≥12 months—57 months) groups, we found that new-onset diabetics possessed significant expansions of MAIT cells among CD8 T cells, T cells, and total leukocytes, yet long-standing diabetics showed no evidence of increasing proportion of MAIT cells with age among any cellular compartment. These data suggest that among new-onset diabetics, MAIT cell expansions are similar to those observed in healthy controls, while MAIT cells from long-standing diabetics do not appear to expand with age. We are currently uncertain as to the cause of these limits observed among long-standing diabetics. Several factors, such as increased gut permeability, altered gut flora, and altered intestinal morphology, may be contributing to our observations. Indeed, several studies have demonstrated an increase in gut permeability among type 1 diabetics [21–23]. This could result in increased exposure to microbes and microbial products within the mucosa, thereby inciting inflammation. In this scenario, the changes we are observing in long-standing T1D could be due to increased rates of apoptosis after bacterial encounter as suggested by Cosgrove and colleagues [45] and/or due to more MAIT cells residing within inflamed mucosa and thus less in circulation.

In animal models, it has been shown that MAIT cell development is reliant upon gut microbiota [46] and several studies have demonstrated that the gut microbiome is altered prior to diabetes onset [13–15] and well as after diagnosis [18–20]. Nevertheless, additional studies are required to determine how gut microbial diversity, not just presence or absence, may impact MAIT cell development among both diabetics and healthy controls. As postulated above, reduced quantity and quality of mucosal surface area may impact MAIT cell development by offering less habitable space for microbial symbionts. Although reduced pancreatic weight and volume have been found in type 1 diabetics [47, 48], we know of no study exhibiting any changes in mucosal surface area or intestinal size among human T1Ds. However, at least one study has demonstrated morphological abnormalities among T1D enterocytes [23]. Furthermore, mucosal responses to insulin among T1D revealed reduced protein production from insulin-deprived type 1 diabetics [49] and disturbed gastrointestinal motility has been associated with T1D [50]. Combined, these results suggest the intestinal environment is altered among T1Ds. This could contribute to alterations in gut flora and, consequently, to MAIT cell abundance.

Our analysis also indicates that MAIT cells from diabetics possess an increased proportion of CD27^-^ cells and this appeared most sharply in diabetics under 11 years of age. Although we are uncertain if the CD27^-^ MAIT cells represents an activated or terminally differentiated phenotype, our analysis of cytokine production from CD27^+/-^ MAIT cells revealed both subsets are capable of producing IFN-γ, TNF-α, and IL-17A, consistent with previous reports. However, the CD27^-^ compartment appears to harbor a greater proportion of IL-17A producers than the CD27^+^ compartment. While the presence and absence of CD27 on MAIT cells has been reported [51, 52], the functional status and ontology of CD27^-^ MAIT cells are unresolved. Nevertheless, Leeansyah and colleagues associated the loss of CD27 expression with a history of activation, consistent with current thinking among human T cell subsets [53]. Thus, CD27^-^ MAIT cells may be further differentiated than their CD27^+^ expressing counterparts, suggesting that type 1 diabetics are experiencing increased activation among the MAIT cell compartment. Assuming that the lack of CD27 expression is associated with terminal differentiation and effector function as it is among other T cell subsets, we can conclude that younger diabetics have increased proportions of terminally differentiated MAIT cells. MAIT cells have been reported to be uniquely activated by microbial-derived vitamin B metabolites [43, 54] which are presented in an MR1-dependent fashion. Thus, activation or increased differentiation among MAIT cells should be microbially-based.

We are currently investigating the mechanistic basis for the transition from CD27^+^ to CD27^-^ among MAIT cells in order to better infer the causes of the increases we observe among younger diabetics. Since we observed a significant correlation between increasing CD27^-^ MAIT cell proportions and increasing HbA1c levels, we cannot exclude the possibility that changes we observed in this compartment are related to hyperglycemia. However, the relative contribution of hyperglycemia to increasing CD27^-^ MAIT cells proportions seems to show a stronger relationship between older diabetics, rather than younger. Considering that hyperglycemic conditions may directly or indirectly affect intestinal permeability, gut microbiotic composition, and MAIT cell maturation, further investigations will be necessary to gauge the relative contribution of these and other potential causes for the MAIT cell alterations observed in diabetics. One further possible outcome of hyperglycemia could involve the cytokine IL-18. It is known that elevated glucose levels have been associated with the release of IL-18 among healthy individuals and those with impaired glucose tolerance [55]. Furthermore, it is know that T1Ds possess increased levels of circulating IL-18 [56, 57], and that IL-18, in combination with IL-12, can strongly activate MAIT cells [58]. These results suggest that early metabolic changes associated with T1D may contribute to the development of terminally differentiated MAIT cells. Together with microbial metabolites, these signals could exert the stimulation necessary to advance the maturation of this cell compartment.

Ultimately, the factors driving the expansion of CD27^-^ MAIT cells among younger patients remain to be determined. Our data suggest a profound MAIT cell differentiation event among young type 1 diabetics, which would imply enhanced exposure to bacterially-derived MAIT cell activating factors. While it appears that this response subsides somewhat with age, further analysis of patient populations will be necessary to understand the dynamics of this subset among diabetics. Furthermore, the results from this study suggest that the analysis of juvenile diabetics should take into consideration time since diagnosis as well as patient age, since different disease-associated patterns are observable based upon alternate patient stratification.

## Supporting Information

S1 File Correlations between duration of type 1 diabetes(T1D) and proportion of MAIT cells. **A**. Correlation of log(%MAIT cells of CD8 T cells) versus months since diagnosis among NT1D and LT1D. **B**. Results of Pearson’s r analysis and linear regression. **C**. Correlation of log(%MAIT cells of total leukocytes) versus month since diagnosis among NT1D and LT1D. **D**. Results of Pearson’s r analysis and linear regression. For both A and C, solid triangles and solid lines represent NT1D, while open squares and dashed lines represent LT1D.
(TIF)

S2 File Correlations between age in years and proportion of CD27± MAIT cells for new-onset (NT1D) and long-standing type 1 diabetics (LT1D). **A**. Correlation of log(%CD27^+^ of MAIT cells) versus age in years among NT1D and LT1D. **B**. Results of Pearson’s r analysis and linear regression. **C**. Correlation of log(%CD27^-^ of MAIT cells) versus age in years among NT1D and LT1D. **D.** Results of Pearson’s r analysis and linear regression. For both A and C, solid triangles and solid lines represent NT1D, while open squares and dashed lines represent LT1D. * = p<0.05
(TIF)
